# An accelerating algorithm for globally solving nonconvex quadratic programming

**DOI:** 10.1186/s13660-018-1764-1

**Published:** 2018-07-16

**Authors:** Li Ge, Sanyang Liu

**Affiliations:** 1 School of Mathematics and Statistics, Xidian University, Xi’an, China; 2 School of Mathematical Sciences, Henan Institute of Science and Technology, Xinxiang, China

**Keywords:** Nonconvex quadratic programming, Global optimization, Linear relaxation approach, Deleting technique, Branch and bound

## Abstract

To globally solve a nonconvex quadratic programming problem, this paper presents an accelerating linearizing algorithm based on the framework of the branch-and-bound method. By utilizing a new linear relaxation approach, the initial quadratic programming problem is reduced to a sequence of linear relaxation programming problems, which is used to obtain a lower bound of the optimal value of this problem. Then, by using the deleting operation of the investigated regions, we can improve the convergent speed of the proposed algorithm. The proposed algorithm is proved to be convergent, and some experiments are reported to show higher feasibility and efficiency of the proposed algorithm.

## Introduction

In this paper, we consider the following nonconvex quadratic programming problem (NQP):

$$\begin{aligned} \mbox{(NQP)}:\quad \textstyle\begin{cases} \min f(y)=c^{T}y+y^{T}Qy \\ \text{s.t.}\quad y\in Y^{0}= \{ y \in R^{n} \mid Ay\leq b \} , \end{cases}\displaystyle \end{aligned}$$

where $Q=(q_{jk})_{n\times n}$ is a real $n\times n$ symmetric matrix; $A=(a_{jk})_{m\times n}$ is a real $m\times n$ matrix, and $c, y\in R^{n}$; $b\in R^{m}$.

The (NQP) is an important class of global optimization problems, and it is not only because they have broad applications in the fields of statistics and design engineering, optimal control, economic equilibria, financial management, production planning, combinatorial optimization, and so on [1, 2], but also because many other nonlinear problems can be transformed into this form [3–7], such as the following linear multiplicative programming (LMP) problem:

$$\begin{aligned} \text{{(LMP)}}:\quad \textstyle\begin{cases} \min f(y)=\sum_{i=1}^{p}(c_{i}^{T}y+d_{i})(e_{i}^{T}y+f_{i}) \\ \text{s.t.} \quad y\in Y^{0}= \{ y \in R^{n} \mid Ay\leq b \} , \end{cases}\displaystyle \end{aligned}$$

where $p\geq 0$, $c_{i}^{T}=(c_{1},c_{2},\ldots,c_{n})$, $e_{i}^{T}=(e _{1},e_{2},\ldots,e_{n})\in R^{n} $; $A=(a_{jk})_{m\times n}$ is a real $m\times n$ matrix, and $d_{i}, f_{i}\in R$, $i=1,2, \ldots,n$; $b\in R^{m}$. Obviously, the (LMP) can be easily transformed into the (NQP). So, the applications of the (NQP) also include all the applications of the (LMP).

In the last decades, many solution methods have been developed for globally solving the (NQP) and its special forms. For example, branch-and-cut algorithm [8] and branch-and-bound algorithm [9] for box-constrained nonconvex quadratic programs; two efficient algorithms for linear constrained quadratic programming (LCQP) by Cambini et al. [10] and Li et al. [11]; branch-and-reduce approach by Gao et al. [12]; finite branch-and-bound approach by Burer and Vandenbussche [13]; branch-and-bound method based on outer approximation and linear relaxation by Al-Khayyal et al. [14]; two simplicial branch-and-bound algorithms by Linderoth [15] and Raber [16]; branch-and-cut algorithm by Sherali et al. [17] and Audet et al. [18], and so on. In addition, a branch-and-bound algorithm for finding a global optimal solution for a nonconvex quadratic program with convex quadratic constraints has been proposed by Cheng Lu et al. [19]; based on the different properties of a quadratic function, five different branch-and-bound approaches for solving the (NQP) have been proposed in Refs. [20–24].

Based on the above-mentioned methods, we will present an accelerating algorithm for effectively solving the (NQP) in this paper. Firstly, we present a new linear relaxation technique, which can be used to construct the linear relaxation programming (LRP) of the (NQP). In addition, we divide the initial feasible region into a number of sub-regions and use deleting techniques to reduce the scope of the investigation. Then, the initial problem (NQP) is converted into a series of linear relaxation programming subproblems. We can prove that the solutions of these subproblems can converge to the global optimal value of the original problem (NQP). Finally, numerical results and comparison with the methods mentioned in recent literature show that our algorithm works as well as or better than those methods.

This paper is organized as follows. In Sect. 2, we describe a new linear relaxation approach for establishing the linear relaxation programming of the (NQP). In Sect. 3, a range deleting technique is given for improving the convergent speed of the proposed algorithm, then a range division and deleting algorithm is proposed and its convergence is proved. Some numerical experiments are reported to show the feasibility and effectiveness of our algorithm in Sect. 4.

## New linear relaxation approach

In this section, we will show how to construct the (LRP) for the (NQP). Let the *i*th row of the matrix *Q* be $Q_{i}$, and let $z_{i}=Q_{i}y= \sum_{k=1}^{n}q_{ik}y_{k}$, $i=1,2,\ldots,n$, we obtain

$$ {z= ({z_{1}},{z_{2}}, \ldots,{z_{n}})^{T}=({Q_{1}}y, {Q_{2}}y, \ldots, {Q_{n}}y)^{T} ={Q}y }. $$

By introducing new variables $z_{i}$, $i=1, 2,\ldots,n$, the function $f(y)$ can be expressed as follows:

$$ f(y,z)=c^{T}y+y^{T}z=\sum_{i=1}^{n}c_{i}y_{i}+ \sum_{i=1} ^{n}y_{i}z_{i}, $$

and the set *D* is defined as

$$ D= \bigl\{ y\mid Ay\le b, y\in R^{n} \bigr\} . $$

We compute the initial variable bounds by solving the following linear programming problems:

$$\begin{aligned}& \underline{y}_{i}^{0}=\min_{y\in D}y,\qquad \overline{y}_{i}^{0}= \max_{y\in D}y, \quad i=1,2,\ldots,n, \\& \underline{z}_{i}^{0}=\min_{y\in D}Q_{i}y, \qquad \overline{z}_{i} ^{0}=\max_{y\in D}Q_{i}y, \quad i=1,2,\ldots,n, \end{aligned}$$

and let

$$\begin{aligned}& Y^{0}= \bigl\{ y \in R^{n} \mid \underline{y}_{i}^{0} \le y_{i} \le \overline{y}_{i}^{0},i=1,2,\ldots,n \bigr\} , \\& Z^{0}= \bigl\{ z \in R^{n} \mid \underline{z}_{i}^{0} \le z_{i} \le \overline{z}_{i}^{0},i=1,2,\ldots,n \bigr\} , \\& \Omega^{0}= \bigl\{ (y,z) \in R^{2n} \mid \underline{y}_{i}^{0} \le y _{i} \le \overline{y}_{i}^{0},\underline{z}_{i}^{0} \le z_{i} \le \overline{z}_{i}^{0},i=1,2,\ldots,n \bigr\} . \end{aligned}$$

Based on the above discussion, we can transform the initial problem into the following equivalent problem (EP):

$$\begin{aligned}& \text{(EP)}:\quad \textstyle\begin{cases} \min f(y,z)=\sum_{i=1}^{n}c_{i}y_{i}+\sum_{i=1}^{n}y _{i}z_{i} \\ \text{s.t.}\quad Ay\leq b, \\ \hphantom{\text{s.t.}\quad} z_{i}=\sum_{k=1}^{n}q_{ik}y_{k},i=1,2,\ldots,n, \\ \hphantom{\text{s.t.}\quad} (y,z) \in \Omega^{0}. \end{cases}\displaystyle \end{aligned}$$

For globally solving the (NQP), the computation of lower bounds for this problem and its subdivided subproblems is the principal operation in the establishment of a branch-and-bound procedure. A lower bound on the optimal value of the (NQP) and its subdivided subproblems can be gotten by solving a linear relaxation programming of the (EP), which can be derived by a new linear relaxation approach.

Let

$$\begin{aligned}& Y=\bigl\{ y\in R^{n}\mid -\infty \leq \underline{y}_{i}\leq y_{i}\leq \overline{y}_{i}\leq +\infty, i=1,2,\ldots,n\bigr\} \subseteq Y^{0}, \\& Z=\bigl\{ z\in R^{n}\mid -\infty \leq \underline{z}_{i}\leq z_{i}\leq \overline{z}_{i}\leq +\infty, i=1,2,\ldots,n\bigr\} \subseteq Z^{0} \end{aligned}$$

for any $y\in Y$, $z\in Z$, $i\in \{1,2,\ldots,n\}$, define the functions

$$\begin{aligned}& \varphi (y_{i})=y^{2}_{i}, \qquad \varphi^{l}(y_{i})=(\underline{y}_{i}+ \overline{y}_{i})y_{i}- \frac{(\underline{y}_{i}+\overline{y}_{i})^{2}}{4}, \\& \varphi^{u}(y_{i})=( \underline{y}_{i}+ \overline{y}_{i})y_{i}-\underline{y}_{i} \overline{y}_{i}, \\& \varphi (z_{i})=z^{2}_{i},\qquad \varphi^{l}(z_{i})=(\underline{z}_{i}+ \overline{z}_{i})z_{i}- \frac{(\underline{z}_{i}+\overline{z}_{i})^{2}}{4}, \\& \varphi^{u}(z_{i})=( \underline{z}_{i}+ \overline{z}_{i})z_{i}-\underline{z}_{i} \overline{z}_{i}, \qquad \varphi (y_{i},z_{i})=y_{i}z_{i}, \\& \varphi^{l}(y_{i},z_{i})= \frac{1}{2}\biggl[(\underline{y}_{i}+\overline{y}_{i})z_{i}+( \underline{z} _{i}+\overline{z}_{i})y_{i}- \frac{(\underline{y}_{i}+\overline{y} _{i}+\underline{z}_{i}+\overline{z}_{i})^{2}}{4}+\underline{y}_{i} \overline{y}_{i}+ \underline{z}_{i}\overline{z}_{i}\biggr], \\& \varphi^{u}(y_{i},z_{i})=\frac{1}{2} \biggl[(\underline{y}_{i}+\overline{y} _{i})z_{i}+( \underline{z}_{i}+\overline{z}_{i})y_{i}- ( \underline{y} _{i}+\underline{z}_{i}) (\overline{y}_{i}+ \overline{z}_{i})+\frac{( \underline{y}_{i}+\overline{y}_{i})^{2}}{4}+\frac{(\underline{z}_{i}+ \overline{z}_{i})^{2}}{4}\biggr]. \end{aligned}$$

Since $\varphi ({y}_{i})={y}_{i}^{2}$ is a convex function of ${y}_{i}$ over $[\underline{{y}_{i}},\overline{{y}_{i}}]$, its affine concave envelope is $\varphi^{u}({y}_{i})=(\underline{{y}_{i}}+\overline{ {y}_{i}}){y}_{i}-\underline{{y}_{i}}\overline{{y}_{i}}$. Moreover, the tangential supporting function for $\varphi ({y}_{i})$ is parallel with the $\varphi^{u}({y}_{i})$, thus the point of tangential support will occur at $\frac{\underline{{y}_{i}}+\overline{{y}_{i}}}{2}$, and the corresponding tangential supporting function is $\varphi^{l}({y}_{i})=(\underline{ {y}_{i}}+\overline{{y}_{i}}){y}_{i}-\frac{(\underline{{y}_{i}}+\overline{ {y}_{i}})^{2}}{4} $. Hence, by the geometric property of the function $\varphi ({y}_{i})$, we have

1 $$ \varphi^{l}({y}_{i})=(\underline{{y}_{i}}+ \overline{{y}_{i}}){y}_{i}-\frac{(\underline{ {y}_{i}}+\overline{{y}_{i}})^{2}}{4}\leq {y}_{i}^{2} \leq (\underline{ {y}_{i}}+ \overline{{y}_{i}}){y}_{i}-\underline{{y}_{i}} \overline{ {y}_{i}}=\varphi^{u}({y}_{i}). $$

Similarly, it follows that

$$ \varphi^{l}(z_{i})\leq \varphi (z_{i}) \leq \varphi^{u}(z_{i}). $$

Furthermore, assume that $({y}_{i}+{z}_{i})$ is a single variable, then $({y}_{i}+{z}_{i})^{2}$ is a convex function about the univariate variable $({y}_{i}+{z}_{i})$ over the interval $[\underline{{y}_{i}}+\underline{ {z}_{i}}, \overline{{y}_{i}}+\overline{{z}_{i}}]$. By the conclusion above, we have

2 $$ (\underline{{y}_{i}}+\underline{{z}_{i}}+ \overline{{y}_{i}}+\overline{ {z}_{i}}) ({y}_{i}+{z}_{i})- \frac{(\underline{{y}_{i}}+\underline{ {z}_{i}}+\overline{{y}_{i}}+\overline{{z}_{i}})^{2}}{4}\leq ({y}_{i}+ {z}_{i})^{2} $$

and

3 $$ ({y}_{i}+{z}_{i})^{2}\leq (\underline{{y}_{i}}+ \underline{{z}_{i}}+\overline{ {y}_{i}}+ \overline{{z}_{i}}) ({y}_{i}+{z}_{i})-( \underline{{y}_{i}}+\underline{ {z}_{i}}) ( \overline{{y}_{i}}+\overline{{z}_{i}}). $$

By (1)–(3), we can obtain

$$\begin{aligned} \varphi (y_{i}, z_{i}) =&\frac{1}{2} \bigl[(y_{i}+z_{i})^{2}-y^{2}_{i}-z ^{2}_{i}\bigr] \\ \geq &\frac{1}{2}\biggl[(\underline{y}_{i}+ \underline{z}_{i}+\overline{y} _{i}+\overline{z}_{i}) (y_{i}+z_{i})-\frac{(\underline{y}_{i}+ \underline{z}_{i}+\overline{y}_{i}+\overline{z}_{i})^{2}}{4}\biggr] \\ &{}-\frac{1}{2}\bigl[(\underline{y}_{i}+\overline{y}_{i})y_{i}- \underline{y}_{i}\overline{y}_{i}+(\underline{z}_{i}+ \overline{z}_{i})z _{i}-\underline{z}_{i} \overline{z}_{i}\bigr] \\ =&\frac{1}{2}\biggl[(\underline{y}_{i}+\overline{y}_{i})z_{i}+( \underline{z}_{i}+\overline{z}_{i})y_{i}- \frac{(\underline{y}_{i}+ \underline{z}_{i}+\overline{y}_{i}+\overline{z}_{i})^{2}}{4}+ \underline{y}_{i}\overline{y}_{i}+ \underline{z}_{i}\overline{z}_{i}\biggr] \\ =&\varphi^{l}(y_{i},z_{i}), \end{aligned}$$

and

$$\begin{aligned} \varphi (y_{i}, z_{i}) =&\frac{1}{2} \bigl[(y_{i}+z_{i})^{2}-y^{2}_{i}-y ^{2}_{k}\bigr] \\ \leq &\frac{1}{2}\bigl[(\underline{y}_{i}+ \underline{z}_{i}+\overline{y} _{i}+\overline{z}_{i}) (y_{i}+z_{i})-(\underline{y}_{i}+\underline{z} _{i}) (\overline{y}_{i}+\overline{z}_{i})\bigr] \\ &{}-\frac{1}{2}\biggl[((\underline{y}_{i}+ \overline{y}_{i})y_{i}-\frac{( \underline{y}_{i}+\overline{y}_{i})^{2}}{4}+( \underline{z}_{i}+ \overline{z}_{i})z_{i}- \frac{(\underline{z}_{i}+\overline{z}_{i})^{2}}{4}\biggr] \\ =&\frac{1}{2}\biggl[(\underline{y}_{i}+\overline{y}_{i})z_{i}+( \underline{z}_{i}+\overline{z}_{i})y_{i}-( \underline{y}_{i}+ \underline{z}_{i}) (\overline{y}_{i}+ \overline{z}_{i})+\frac{( \underline{y}_{i}+\overline{y}_{i})^{2}}{4} +\frac{(\underline{z}_{i}+\overline{z}_{i})^{2}}{4}\biggr] \\ =&\varphi^{u}(y_{i},z_{i}). \end{aligned}$$

Hence, we have

4 $$ \varphi^{l}(y_{i}, z_{i})\leq \varphi (y_{i}, z_{i})\leq \varphi^{u}(y _{i},z_{i}). $$

Furthermore, we define the difference functions as follows:

$$\begin{aligned}& \triangle (y_{i})=\varphi (y_{i})-\varphi^{l}(y_{i}), \qquad \nabla (y _{i})=\varphi^{u}(y_{i})- \varphi (y_{i}), \\& \triangle (z_{i})=\varphi (z_{i})-\varphi^{l}(z_{i}), \qquad \nabla (z _{i})=\varphi^{u}(z_{i})-\varphi (z_{i}), \\& \triangle (y_{i},z_{i})=\varphi (y_{i}, z_{i})-\varphi ^{l}(y_{i}, z_{i}), \qquad \nabla (y_{i},z_{i})=\varphi^{u}(y_{i},z_{i})- \varphi (y_{i}, z_{i}). \end{aligned}$$

Since $\triangle (y_{i})$ is convex about $y_{i}$, for any $y_{i} \in [\underline{{y}_{i}},\overline{{y}_{i}}]$, it follows that $\triangle (y_{i})$ can attain the maximum at the point $\underline{y _{i}}$ or $\overline{y_{i}}$. Then

$$ \max_{y_{i}\in [\underline{{y}_{i}},\overline{{y}_{i}}]}\triangle (y _{i})=\varphi ( \overline{y_{i}})-\varphi^{l}(\overline{y_{i}})= \varphi (\underline{y_{i}})-\varphi^{l}(\underline{y_{i}})= \frac{(\overline{y _{i}}-\underline{y_{i}})^{2}}{4}. $$

On the other hand, since $\nabla (y_{i})$ is concave about $y_{i}$, for any $y_{i}\in [\underline{{y}_{i}},\overline{{y}_{i}}]$, $\nabla (y _{i})$ can attain the maximum at the point $\frac{\underline{y_{i}}+\overline{y _{i}}}{2}$, i.e.,

$$ \max_{y_{i}\in [\underline{{y}_{i}},\overline{{y}_{i}}]}\nabla (y_{i})= \varphi^{u} \biggl(\frac{\underline{y_{i}}+\overline{y_{i}}}{2}\biggr)-\varphi \biggl(\frac{\underline{y _{i}}+\overline{y_{i}}}{2}\biggr)= \frac{(\overline{y_{i}}-\underline{y_{i}})^{2}}{4}. $$

So, we have

5 $$ \max_{y_{i}\in [\underline{y}_{i}, \overline{y}_{i}]}\triangle (y_{i})= \max _{y_{i}\in [\underline{y}_{i}, \overline{y}_{i}]}\nabla (y_{i}) \rightarrow 0, \quad \text{as } \vert \overline{y}_{i}-\underline{y}_{i} \vert \rightarrow 0, $$

that is, $\triangle (y_{i})$, $\nabla (y_{i})\rightarrow 0$, as $\|\overline{y}-\underline{y}\|\rightarrow 0$.

Similarly, we can prove that

6 $$ \max_{z_{i}\in [\underline{z}_{i}, \overline{z}_{i}]}\triangle (z_{i})= \max _{z_{i}\in [\underline{z}_{i}, \overline{z}_{i}]}\nabla (z_{i}) \rightarrow 0, \quad \text{as } \vert \overline{z}_{i}-\underline{z}_{i} \vert \rightarrow 0, $$

and $\triangle (z_{i})$, $\nabla (z_{i})\rightarrow 0$, as $\| \overline{z}-\underline{z}\|\rightarrow 0$.

Define

$$\begin{aligned}& \triangle (y_{i}+z_{i})=(y_{i}+z_{i})^{2}- \biggl[(\underline{y}_{i}+ \overline{y}_{i}+ \underline{z}_{i}+\overline{z}_{i}) (y_{i}+z_{i})- \frac{( \underline{y}_{i}+\overline{y}_{i}+\underline{z}_{i}+\overline{z}_{i})^{2}}{4}\biggr], \\& \nabla (y_{i}+z_{i})=(\underline{y}_{i}+ \overline{y}_{i}+ \underline{z}_{i}+\overline{z}_{i}) (y_{i}+z_{i})-(\underline{y}_{i}+ \underline{z}_{i}) (\overline{y}_{i}+\overline{z}_{i})-(y_{i}+z_{i})^{2}. \end{aligned}$$

Using the similar method, we can get the following conclusions:

7 $$\begin{aligned}& \max_{(y_{i}+z_{i})\in [(\underline{y}_{i}+\underline{z}_{i}),( \overline{y}_{i}+\overline{z}_{i})]}\triangle (y_{i}+z_{i}) \\& \quad = \max_{(y_{i}+z_{i})\in [(\underline{y}_{i}+\underline{z}_{i}), ( \overline{y}_{i}+\overline{z}_{i})]}\nabla (y_{i}+z_{i}) \rightarrow 0,\quad \text{as } \Vert \overline{y}-\underline{y} \Vert \rightarrow 0, \Vert \overline{z}-\underline{z} \Vert \rightarrow 0. \end{aligned}$$

Since

$$\begin{aligned} \triangle (y_{i},z_{i}) =&\varphi (y_{i}, z_{i})-\varphi^{l}(y_{i}, z _{i}) \\ =&y_{i}z_{i}-\frac{1}{2}\biggl[( \underline{y}_{i}+\overline{y}_{i})z_{i}+( \underline{z}_{i}+\overline{z}_{i})y_{i}- \frac{(\underline{y}_{i}+ \overline{y}_{i}+\underline{z}_{i}+\overline{z}_{i})^{2}}{4}+ \underline{y}_{i}\overline{y}_{i}+ \underline{z}_{i}\overline{z}_{i}\biggr] \\ =&\frac{1}{2}\bigl[(y_{i}+z_{i})^{2}-y^{2}_{i}-y^{2}_{k} \bigr] \\ &{}-\frac{1}{2}\biggl[(\underline{y}_{i}+ \overline{y}_{i})z_{i}+( \underline{z}_{i}+ \overline{z}_{i})y_{i}-\frac{(\underline{y}_{i}+ \overline{y}_{i}+\underline{z}_{i}+\overline{z}_{i})^{2}}{4}+ \underline{y}_{i}\overline{y}_{i}+\underline{z}_{i} \overline{z}_{i}\biggr] \\ =&\frac{1}{2}\biggl[(y_{i}+z_{i})^{2}- \biggl((\underline{y}_{i}+\overline{y}_{i}+ \underline{z}_{i}+\overline{z}_{i}) (y_{i}+z_{i})- \frac{(\underline{y} _{i}+\overline{y}_{i}+\underline{z}_{i}+\overline{z}_{i})^{2}}{4}\biggr)\biggr] \\ &{}+\frac{1}{2}\bigl[\bigl((\underline{y}_{i}+ \overline{y}_{i})y_{i}- \underline{y}_{i} \overline{y}_{i}-y^{2}_{i}\bigr)+\bigl(( \underline{z}_{i}+ \overline{z}_{i})z_{i}- \underline{z}_{i}\overline{z}_{i}-z^{2}_{i} \bigr)\bigr] \\ =&\frac{1}{2}\triangle (y_{i}+z_{i})+ \frac{1}{2}\nabla (y_{i})+ \frac{1}{2}\nabla (z_{i}) \\ \leq &\frac{1}{2} \max_{(y_{i}+z_{i})\in [(\underline{y}_{i}+\underline{z}_{i}), ( \overline{y}_{i}+\overline{z}_{i})]}\triangle (y_{i}+z_{i}) +\frac{1}{2}\max_{y_{i}\in [\underline{y}_{i}, \overline{y}_{i}]} \nabla (y_{i})+ \frac{1}{2} \max_{z_{i}\in [\underline{z}_{i}, \overline{z}_{i}]}\nabla (z_{i}), \end{aligned}$$

by (5)–(7), we can get that

8 $$ \triangle (y_{i},z_{i})\rightarrow 0, \quad \text{as } \Vert \overline{y}- \underline{y} \Vert \rightarrow 0, \Vert \overline{z}- \underline{z} \Vert \rightarrow 0. $$

Similarly, we can prove that $\nabla (y_{i},z_{i})\rightarrow 0$, as $\|\overline{y}-\underline{y}\|\rightarrow 0$, $\| \overline{z}-\underline{z}\|\rightarrow 0$.

For convenience, without loss of generality, for any $y\in Y\subseteq Y^{0}$, $z\in Z\subseteq Z^{0}$, define

$$ f^{L}(y,z)=\sum_{i=1}^{n}c_{i}y_{i}+ \sum_{i=1}^{n} \varphi^{l}(y_{i}, z_{i}). $$

By utilizing the convexity of a univariate quadratic function, we establish an effective method for generating the linear underestimation and linear overestimation of the functions $y^{2}_{i}$, $z^{2}_{i}$, and $y_{i}z_{i}$, respectively. By the conclusions above, the linear relaxation underestimation function $f^{L}(y,z)$ of the function $f(y,z)$ for the (EP) can be established. Thus, by the former discussions, we can construct the corresponding approximation linear relaxation programming (LRP) of the (EP) as follows:

$$\begin{aligned} \text{{(LRP)}}: \quad \textstyle\begin{cases} \min f^{L}(y,z)=\sum_{i=1}^{n}c_{i}y_{i}+\sum_{i=1} ^{n}\varphi^{l}(y_{i}, z_{i}) \\ \text{s.t.}\quad Ay\leq b, \\ \hphantom{\text{s.t.}\quad}z_{i}=\sum_{k=1}^{n}q_{ik}y_{k},i=1,2,\ldots,n, \\ \hphantom{\text{s.t.}\quad} (y,z) \in \Omega^{0}. \end{cases}\displaystyle \end{aligned}$$

By (4), it is obvious that

$$ y_{i}z_{i}-{\varphi }^{l}(y_{i},z_{i}) \geq 0, $$

then

$$\begin{aligned}& f(y,z)-f^{L}(y,z) \\& \quad =\sum_{k=1}^{n}c_{i}y_{i}+ \sum_{k=1} ^{n}y_{i}z_{i}- \Biggl[\sum_{k=1}^{n}c_{i}y_{i}+ \sum_{k=1}^{n} {\varphi }^{l}(y_{i}, z_{i})\Biggr] \\& \quad=\sum_{k=1}^{n}\bigl[y_{i}z_{i}-{ \varphi }^{l}(y_{i}, z_{i})\bigr] \\& \quad \geq 0. \end{aligned}$$

Thus, we have $f(y,z)-f^{L}(y,z)\geq 0$, i.e., $f(y,z)\geq f^{L}(y,z)$.

Furthermore, we have

$$\begin{aligned} f(y,z)-f^{L}(y,z) =&\sum_{k=1}^{n} \bigl[y_{i}z_{i}-{\varphi }^{l}(y _{i}, z_{i})\bigr] \\ =&\sum_{k=1}^{n}\triangle (y_{i},z_{i}). \end{aligned}$$

By (8), we have that $\triangle (y_{i},z_{i})\rightarrow 0$, as $\|\overline{y}-\underline{y}\|\rightarrow 0$, $\|\overline{z}- \underline{z}\|\rightarrow 0$.

Therefore, we have that

$$ f(y,z)-f^{L}(y,z)\rightarrow 0 \quad \text{as } \Vert \overline{y}- \underline{y} \Vert \rightarrow 0, \Vert \overline{z}-\underline{z} \Vert \rightarrow 0. $$

### Remark 1

From above, we only need to solve the (LRP) instead of solving the (EP) to obtain the lower and upper bounds of the optimal value in problem (NQP).

### Remark 2

Based on the construction of the (LRP), each feasible point of the (NQP) in the sub-range Ω is also feasible to LRP, and the global minimum value of LRP is not more than that of the (NQP) in the sub-range Ω. Thus, the (LRP) can provide a valid lower bound for the global optimum value of problem NQP in the sub-range Ω.

### Remark 3

The conclusions above ensure that the linear relaxation programming LRP can infinitely approximate the (NQP), as $\|Y\|\rightarrow 0$ (obviously, $\|Z\|\rightarrow 0$), this will guarantee the global convergence of the proposed algorithm.

## Accelerating branch-and-bound algorithm and its convergence

Now we establish an accelerating branch-and-bound algorithm based upon the former linear relaxation approach for globally solving the (NQP). We will introduce the algorithm process at first and then give the convergence analysis of the algorithm.

### Branching process

The critical operation of the branching process is iteratively subdividing the initial range $\Omega^{0}$ into some sub-ranges, each sub-range denoted by Ω is concerned with a node of the branch and bound, such that any infinite iterative sequence of partition sets shrinks to a singleton. Here, we will adopt a standard range bisection approach, which is adequate to ensuring global convergence of the proposed algorithm. Detailed process is described as follows.

For any selected sub-range $\Omega^{\prime}\in \Omega^{0}$, $Y^{\prime}=[ \underline{y}^{\prime},\overline{y}^{\prime}]\subseteq Y^{0}$, let $q\in \arg \max \{\overline{y}_{i}^{\prime}-\underline{y}_{i}^{\prime}: i=1,2,\ldots,n\}$, and $y_{m}=(\underline{y}_{q}^{\prime}+\overline{y}_{q}^{\prime})/2$, then divide $\Omega^{\prime}$ into two sub-ranges $\Omega^{\prime}_{1}$ and $\Omega^{\prime}_{2}$ by subdividing the interval $[\underline{y}^{\prime}_{q},\overline{y}^{\prime} _{q}]$ into two subintervals $[\underline{y}_{q}^{\prime},y_{m}]$ and $[y_{m},\overline{y}_{q}^{\prime}]$.

From the above branching operation, we can notice that the intervals $[\underline{z}^{\prime},\overline{z}^{\prime}]$ of *z* will never be partitioned by the branching processes. Hence, these branching operations only occur in a space of dimension *n*, i.e., the proposed algorithm economizes the required computations.

### Range deleting technique

At the *k*th iteration of the algorithm, for any rectangle $\Omega ^{k}\subseteq \Omega^{0}$, we want to check whether $\Omega^{k}$ contains a global optimal solution of the $(\mathrm{EP})(\Omega^{0})$, where

$$ {{\Omega }^{k}}=\Omega_{1}^{k}\times \Omega_{2}^{k}\times \cdots \times \Omega_{q-1}^{k} \times \Omega_{q}^{k}\times \Omega_{q+1}^{k} \times \cdots \times \Omega_{n}^{k}, $$

with

$$ \Omega_{q}^{k}=\bigl\{ ({{y}_{q}},{{z}_{q}}) \in {{R}^{2}}\mid \underline{y} _{q}^{k}\leq {y}_{q}^{k}\leq \overline{y}_{q}^{k}, \underline{z}_{q} ^{0}\leq {z}_{q}^{0} \leq \overline{z}_{q}^{0}\bigr\} . $$

#### Theorem 3.1

*Assume that* *f̅* *is a known upper bound of the optimal value* *v* *of the* (*EP*), *for any sub*-*range* $\Omega^{k} \subseteq \Omega^{0}$, *the following conclusions hold*: (*i*) *If* $ELB^{k}>\overline{f}$, *then there is no global optimal solution for the* (*EP*) *over* $\Omega^{k}$; (*ii*) *If* $ELB^{k}\le \overline{f} $, *then we have*: *for any* $\tau \in \{1,2, \ldots,n\}$, *if* $c_{\tau }>0$, *then the sub*-*range* $\overline{\Omega }^{k} $ *does not contain any global optimal solution of the* (*EP*); *else if* $c_{\tau }<0$, *then the sub*-*range* $\overline{\overline{\Omega }} ^{k} $ *does not contain any global optimal solution of the* (*EP*), *where*

$$\begin{aligned}& ELB^{k}=\sum_{i=1}^{n} \min \bigl\{ c_{i}\underline{y}_{i}^{k},c_{i} \overline{y}_{i}^{k}\bigr\} + \sum _{i=1}^{n}\min \bigl\{ \underline{y}_{i} ^{k}\underline{z}_{i}^{0},\underline{y}_{i}^{k} \overline{z}_{i}^{0}, \overline{y}_{i}^{k} \underline{z}_{i}^{0},\overline{y}_{i}^{k} \overline{z}_{i}^{0}\bigr\} , \\& \rho_{\tau }^{k}=\frac{\overline{f}-RLB^{k}+\min \{c_{\tau } \underline{y}_{\tau }^{k},c_{\tau }\overline{y}_{\tau }^{k}\}}{c_{ \tau }}, \quad \tau =1,\ldots,n, \\& \overline{\Omega }^{k}=\Omega_{1}^{k}\times \Omega_{2}^{k}\times \cdots \times \Omega_{\tau -1}^{k} \times \overline{\Omega } _{\tau } ^{k}\times \Omega_{\tau +1}^{k}\times \cdots \times \Omega_{n}^{k}, \\& \overline{\overline{\Omega }}^{k}=\Omega_{1}^{k} \times \Omega_{2}^{k} \times \cdots \times \Omega_{\tau -1}^{k}\times \overline{\overline{ \Omega }}_{\tau }^{k}\times \Omega_{\tau +1}^{k}\times \cdots \times \Omega_{n}^{k}, \end{aligned}$$

*with*

$$\begin{aligned}& \overline{\Omega }_{\tau }^{k}=\bigl\{ ({{y}_{\tau }},{{z}_{\tau }}) \in {{R}^{2}}\mid \rho_{\tau }^{k}\leq {y}_{\tau }^{k}\leq \overline{y} _{\tau }^{k}, \underline{z}_{\tau }^{0}\leq {z}_{\tau }^{0} \leq \overline{z}_{\tau }^{0}\bigr\} \cap {\Omega } _{\tau }^{k}, \\& \overline{\overline{\Omega }}_{\tau }^{k}=\bigl\{ ({{y}_{\tau }},{{z}_{ \tau }})\in {{R}^{2}}\mid \underline{y}_{\tau }^{k}\leq {y}_{\tau } ^{k} \leq \rho_{\tau }^{k}, \underline{z}_{\tau }^{0} \leq {z}_{\tau } ^{0}\leq \overline{z}_{\tau }^{0} \bigr\} \cap {\Omega } _{\tau }^{k}. \end{aligned}$$

#### Proof

(i) If $ELB^{k}>\overline{f}$, then for all $(y,z) \in \Omega^{k}$,

$$\begin{aligned} f(y,z) = & \sum_{i=1}^{n}c_{i}y_{i}+ \sum_{i=1}^{n}y _{i}z_{i} \\ \ge & \sum_{i=1}^{n}\min \bigl\{ c_{i}\underline{y}_{i}^{k},c_{i} \overline{y}_{i}^{k}\bigr\} + \sum _{i=1}^{n}\min \bigl\{ \underline{y}_{i} ^{k}\underline{z}_{i}^{0},\underline{y}_{i}^{k} \overline{z}_{i}^{0}, \overline{y}_{i}^{k} \underline{z}_{i}^{0},\overline{y}_{i}^{k} \overline{z}_{i}^{0}\bigr\} \\ =& ELB^{k}>\overline{f}. \end{aligned}$$

Therefore, there is no global optimal solution for the (EP) over $\Omega^{k}$.

(ii) If $ELB^{k}\le \overline{f}$, for any $\tau \in \{1,2,\ldots,n\}$, then consider the following two cases.

Case 1: If $c_{\tau }>0$, for all $(y,z) \in \overline{\Omega }^{k} $, we have $y_{\tau }>{\rho_{\tau }}$, i.e., $c_{\tau }y_{\tau }> \overline{f}-ELB^{k}+\min \{c_{\tau }\underline{y}_{\tau }^{k}, c_{ \tau }\overline{y}_{\tau }^{k}\}$. Furthermore, we can get that

$$\begin{aligned} f(y,z) =&\sum_{i=1,i\neq \tau }^{n}c_{i}y_{i}+c_{\tau }y_{ \tau }+ \sum_{i=1}^{n}y_{i}z_{i} \\ \geq & \sum_{i=1,i\neq \tau }^{n}\min \bigl\{ c_{i}\underline{y} _{i}^{k}, c_{i} \overline{y}_{i}^{k}\bigr\} +c_{\tau }y_{\tau }+ \sum_{i=1}^{n}\min \bigl\{ \underline{y}_{i}^{k}\underline{z}_{i}^{0}, \underline{y}_{i}^{k}\overline{z}_{i}^{0}, \overline{y}_{i}^{k} \underline{z}_{i}^{0}, \overline{y}_{i}^{k}\overline{z}_{i}^{0} \bigr\} \\ >&\sum_{i=1,i\neq \tau }^{n}\min \bigl\{ c_{i}\underline{y}_{i}^{k}, c_{i} \overline{y}_{i}^{k}\bigr\} +\sum _{i=1}^{n}\min \bigl\{ \underline{y} _{i}^{k}\underline{z}_{i}^{0}, \underline{y}_{i}^{k}\overline{z}_{i} ^{0}, \overline{y}_{i}^{k}\underline{z}_{i}^{0}, \overline{y}_{i}^{k} \overline{z}_{i}^{0} \bigr\} +\overline{f} \\ &{}-ELB^{k}+\min \bigl\{ c_{\tau }\underline{y}_{\tau }^{k}, c_{\tau } \overline{y}_{\tau }^{k}\bigr\} \\ =&ELB^{k}+\overline{f}-ELB^{k} \\ =&\overline{f}. \end{aligned}$$

Thus, we have

$$ f(y,z)>\overline{f}. $$

Therefore, the range ${\overline{\Omega }}^{k} $ does not contain any global optimal solution of the (EP).

Case 2: If $c_{\tau }<0$, then for any $(y,z)\in \overline{\overline{ \Omega }}^{k}$, we have $y_{\tau }<\rho_{\tau }$, i.e., $c_{\tau }y _{\tau }>\overline{f}-ELB^{k}+\min \{c_{\tau }\underline{y}_{\tau } ^{k}, c_{\tau }\overline{y}_{\tau }^{k}\}$.

Similar to the proof of Case 1, we can get that

$$ f(y,z)> \overline{f}, $$

thus the range $\overline{\overline{\Omega }}^{k}$ does not contain any global optimal solution of the (EP). Thus, the proof is completed. □

According to Theorem 3.1, we can construct the following range reduction technique to reject the whole investigated sub-range $\Omega^{k}$ or delete a part of the investigated sub-range $\Omega^{k}$ which does not contain any global optimal solution of the (EP).

*Range deleting algorithm*

Calculate $ELB^{k} $, if $ELB^{k}>\overline{f}$, then let $\Omega ^{k}=\emptyset $. Otherwise, for each $\tau \in \{1,\ldots,n\}$, if $c_{\tau }>0$ and $\rho_{\tau }^{k}<\overline{y}_{\tau }^{k}$, then let $\overline{y}_{\tau }^{k}=\rho_{\tau }^{k}$ and $\overline{\Omega } ^{k}$ with $\overline{\Omega }_{\tau }^{k}=\{({{y}_{\tau }},{{z}_{ \tau }})\in {{R}^{2}}\mid \underline{y}_{\tau }^{k}\leq {y}_{\tau } ^{k}\leq \overline{y}_{\tau }^{k}, \underline{z}_{\tau }^{0}\leq {z}_{\tau }^{0}\leq \overline{z}_{\tau }^{0}\}$; else if $c_{\tau }<0$ and $\rho_{\tau }^{k}>\underline{y}_{\tau }^{k}$, then let $\underline{y}_{\tau }^{k}=\rho_{\tau }^{k}$ and $\overline{\Omega } ^{k}$ with $\overline{\Omega }_{\tau }^{k}=\{({{y}_{\tau }},{{z}_{ \tau }})\in {{R}^{2}}\mid \underline{y}_{\tau }^{k}\leq {y}_{\tau } ^{k}\leq \overline{y}_{\tau }^{k}, \underline{z}_{\tau }^{0}\leq {z}_{\tau }^{0}\leq \overline{z}_{\tau }^{0}\}$.

### Branch-and-bound algorithm

Assume that we have gotten the set of active nodes $\Lambda_{k}$ at the *k*th iteration of the algorithm, and each node is associated with a sub-range $\Omega \subseteq \Omega^{0}$ for all $\Omega \in \Lambda _{k}$. For each sub-range Ω, use the proposed range deleting technique to compress the sub-range Ω, still denote the remaining sub-range by Ω, and obtain a lower bound $LB(\Omega)$ of the optimum value of the (NQP) by solving the (LRP) in Ω.

Let $f_{r}(\Omega)$ and $(y_{r}(\Omega),z_{r}(\Omega))$ be the optimal value and the optimal solution of the (LRP) over the sub-range Ω, respectively. Combining the former linear relaxation method, branching process, and range deleting technique, the basic steps of the proposed accelerating algorithm for globally solving the (NQP) may be stated as follows.

*Algorithm statement*

*Initialization step.* Let the initial iteration counter $k:=0$, the initial set of active nodes $\Lambda_{0}=\{\Omega^{0}\}$, the initial upper bound $\overline{f}=+\infty $, the convergent error $\varepsilon >0$, and the initial collection of feasible points $F:=\emptyset $.

Solve the (LRP) over $\Omega^{0}$, obtain its optimal solution $y^{0}:=y_{r}(\Omega^{0})$, $z^{0}:=z_{r}(\Omega^{0})$ and optimal objective function value $LB_{0}:=f_{r}(\Omega^{0})$. If $(y^{0},z ^{0})$ is feasible to the (NQP), then let $\overline{f}=f(y^{0},z^{0})$ and $F=F\cup \{(y^{0},z^{0})\}$. If $\overline{f}-LB_{0}\leq \varepsilon $, then algorithm stops with $(y^{0},z^{0})$ as an *ε*-global optimum solution for the (NQP). Otherwise, proceed with the following *Range deleting step.*

*Range deleting step.* For each sub-range Ω, use the proposed range deleting technique in Sect. 3.2 to contract each sub-range Ω and still denote the remaining sub-range by Ω.

*Range division step.* Apply the presented range division approach to subdivide $\Omega^{k}$ into two new sub-ranges, and denote the collection of new subdivided sub-ranges by $\widehat{\Omega }^{k}$.

*Renewing the lower and upper bound step.* If $\widehat{\Omega } ^{k}\ne \emptyset $ for each $\Omega \in \widehat{\Omega }^{k}$, then solve the $\operatorname{LRP}(\Omega)$ to get $f_{r}(\Omega)$ and $(y_{r}(\Omega),z _{r}(\Omega))$. Let $LB(\Omega):=f_{r}(\Omega)$, if $LB(\Omega)> \overline{f}$, set $\widehat{\Omega }^{k}:=\widehat{\Omega }^{k} \setminus \Omega $. The residual subdivided set is now $\Lambda_{k}:=( \Lambda_{k}\setminus \Omega^{k})\cup \widehat{\Omega }^{k}$, and renew the lower bound $LB_{k}:=\inf_{\Omega \in \Lambda_{k}}LB (\Omega)$.

If the midpoint $y_{\mathrm{mid}}$ of *Y* is feasible to problem (NQP), set $z_{\mathrm{mid}}=Qy_{\mathrm{mid}}$, then let $F:=F\cup \{(y_{\mathrm{mid}},z_{\mathrm{mid}})\}$. Furthermore, if $y_{r}(\Omega)$ is feasible to problem (NQP), then let $F:=F\cup \{(y_{r}(\Omega)),z_{r}(\Omega)\}$.

If $F\ne \emptyset $, renew the upper bound $\overline{f}:= \min_{(y,z)\in F}f(y,z)$, and denote the known best feasible solution by $(y_{\mathrm{best}},z_{\mathrm{best}}):=\operatorname{argmin}_{(y,z)\in F}f(y,z)$.

*Termination checking step.* If $\overline{f}-LB_{k}\le \varepsilon $, the algorithm ends with *f̅* and $y_{\mathrm{best}}$ as the *ε*-global optimum value and a global optimal solution of problem (NQP). Otherwise, set $k:=k+1$ and pick out an active node $\Omega^{k+1}$ satisfying $\Omega^{k+1}=\operatorname{argmin}_{\Omega \in \Lambda_{k}}LB (\Omega)$, and return to *Range deleting step.*

### Convergence analysis

In this subsection, the convergence of this algorithm is discussed as follows.

#### Theorem 3.2

*Suppose that the feasible region* *D* *of the* (*NQP*) *is nonempty*, *then the above algorithm either terminates finitely with a global optimum solution of the* (*NQP*), *or generates an infinite sequence* $\{y^{k}\}$ *of which any accumulation point will be a global optimal solution of the* (*NQP*).

#### Proof

If the algorithm terminates finitely, it terminates at the $k{th}$ iteration, where $k\geq 0$. Upon termination, by steps in the algorithm, we get that $\overline{f}-LB_{k}\leq \varepsilon $. By the first five steps in the algorithm, there exists a feasible solution $y^{*}$ for the (NQP), which satisfies that $\overline{f}=f(y^{*})$, and $f(y^{*})-LB_{k}\leq \varepsilon $. According to the algorithm, we have that $LB_{k}\leq v$. Since $y^{*}$ is a feasible solution of the (NQP), we have $f(y^{*})\geq v$. Combining the above inequalities, we get that $v\leq f(y^{*})\leq LB_{k}+\varepsilon \leq v+\varepsilon$, i.e., $v\leq f(y^{*})\leq v+\varepsilon$. Therefore, $y^{*}$ is a global *ε*-optimum solution for the (NQP).

If the algorithm is infinite, according to the algorithm, we have that $\{LB_{k}\}$ is a nondecreasing sequence and it has an upper bound $\min_{y\in D}f(y)$, which ensures the existence of the limit $LB:=\lim_{k\rightarrow \infty }LB_{k}\leq \min_{y\in D}f(y)$. Since the sequence $\{y^{k}\}$ is included in a compact set $Y^{0}$, there must exist a convergent subsequence $\{y^{s}\}\subseteq \{y^{k}\}$ and assume that $\lim_{s\rightarrow \infty }y^{s}=y^{*}$. Then by the branching process and deleting method, there must exist a decreasing subsequence $\{Y^{r}\}\subset Y^{s}$, where $(Y^{s},Z^{s})\in \Lambda_{s}$ with $y^{r}\in Y^{r}$, $LB_{r}=LB(Y^{r})=f^{L}(y^{r})$ and $\lim_{r\rightarrow \infty }Y^{r}=\{y^{*}\}$. By the continuity of function $\Psi_{0}(y)$, we have

$$ \begin{aligned}\lim_{r\rightarrow \infty }LB_{r}&=\lim_{r\rightarrow \infty }f^{L} \bigl(y ^{r}\bigr) \\ &=\lim_{r\rightarrow \infty }f\bigl(y^{r} \bigr) \\ &=f\bigl(y^{*}\bigr). \end{aligned}$$

Then, since $Y^{0}$ is a closed set and $\{y^{k}\}\subset Y^{0}$, obviously, we have that $y^{*}\in Y^{0}$, i.e., $y^{*}$ is a feasible solution of the (NQP). Thus, the proof is completed. □

## Numerical experiments

To verify the performance of the proposed algorithm, some numerical experiments are reported and compared with the known methods [19–25]. The algorithm is implemented by Matlab 2016a, and the tests are run in a microcomputer with Intel(R) Xeon(R) processor of 2.4 GHz, 4 GB of RAM memory, under the Win10 operational system. We used linprog solver to solve all linear programming problems, and the convergent error is set to *ε* in our procedure. For these test examples, the numerical results compared with the current algorithms are demonstrated in Tables 1 and 2, where the following notations have been used in column headers: Opt.Val.: optimal value; Opt.Sol.: optimal solution; Iter.: the number of iterations.

**Table 1:** Numerical comparisons for test Examples 1–6

E.g.	Methods	Opt. val.	Opt. sol.	Iter.	Time (s)
1	ours	10.00000	(2.0000, 8.0000)	3	0.35725
	[25]	10.0000090	(1.9999998, 7.9999988)	41	0.02
	[26]	10	(2, 8)	27	10.83
	[27]	10	(2, 8)	53	0.3
2	ours	3.00000	(0.0000, 4.0000)	8	0.01791
	[28]	3.00000	(0.0000, 4.0000)	25	0.750
3	ours	0.890185	(1.3148, 0.1396, 0.0, 0.4233)	1	0.38982
	[27]	0.8902	(1.3148, 0.1396, 0.0, 0.4233)	1	0.1880
	[29]	0.890193	(1.314792, 0.13955, 0.0, 0.423286)	6	
4	ours	−16.22662	(0.0, 3.6403, 0.0, 2.9029, 1.9388, 0.0)	5	0.59005
5	ours	−3.00000	(3.0000, 3.0000)	30	4.15970
6	ours	−1.06250	(0.7500, 2.0000)	3	0.26845

**Table 2:** Numerical comparisons with Ref. [12, 30] for Example 7

Dimension	Methods	Opt. val.	Iter.	Time (s)
*n* = 5	[12]	−25.0	141	10.11
	[30]	−25.0	12	0.0187
	ours	−25.0	1	0.01254
*n* = 10	[12]	−100.0	283	21.86
	[30]	−100.0	31	0.3342
	ours	−100.0	7	0.25649
*n* = 20	[12]	−400.0	651	47.00
	[30]	−400.0	86	5.9396
	ours	−400.0	15	2.73556
*n* = 30	[12]	−900.0	965	106.33
	[30]	−900.0	204	44.8577
	ours	−900.0	18	11.25635
*n* = 50	[12]	−2500.0	1891	304.30
	ours	−2500.0	21	17.35219
*n* = 80	ours	−6400.0	37	40.45623
*n* = 100	ours	−10,000.0	51	44.35865
*n* = 150	ours	−22,500.0	66	112.99298

### Example 1

(Refs. [25–27])

$$ \textstyle\begin{cases} \min (y_{1}+y_{2})(y_{1}-y_{2}+7) \\ \text{s.t.} \quad 2y_{1}+y_{2}\leq 14, \\ \hphantom{\text{s.t.} \quad} y_{1}+y_{2}\leq 10, \\ \hphantom{\text{s.t.} \quad}-4y_{1}+y_{2}\leq 0, \\ \hphantom{\text{s.t.} \quad} 2y_{1}+y_{2}\geq 6, \\ \hphantom{\text{s.t.} \quad} y_{1}+2y_{2}\geq 6, \\ \hphantom{\text{s.t.} \quad} y_{1}-y_{2}\leq 3, \\ \hphantom{\text{s.t.} \quad} y_{1}\leq 5, \\ \hphantom{\text{s.t.} \quad} y_{1}+y_{2}\geq 0, \\ \hphantom{\text{s.t.} \quad}y_{1}-y_{2}+7\geq 0. \end{cases} $$

### Example 2

(Ref. [28])

$$ \textstyle\begin{cases} \min y_{1}+(2y_{1}-3y_{2}+13)(y_{1}+y_{2}-1) \\ \text{s.t.}\quad -y_{1}+2y_{2}\leq 8, \\ \hphantom{\text{s.t.} \quad} -y_{2}\leq -3, \\ \hphantom{\text{s.t.} \quad} y_{1}+2y_{2}\leq 12, \\ \hphantom{\text{s.t.} \quad} y_{1}-2y_{2}\leq -5, \\ \hphantom{\text{s.t.} \quad} y_{1},y_{2}\geq 0. \end{cases} $$

### Example 3

(Refs. [27, 29])

$$ \textstyle\begin{cases} \min (0.813396y_{1}+0.67440y_{2}+0.305038y_{3}+0.129742y_{4}+0.217796) \\ \quad {}\times (0.224508y_{1}+0.063458y_{2}+0.932230y_{3}+0.528736y_{4}+0.091947) \\ \text{s.t.}\quad 0.488509y_{1} +0.063565y_{2} +0.945686y_{3}+ 0.210704y _{4} \leq 3.562809, \\ \hphantom{\text{s.t.}\quad} {-}0.324014y_{1} -0.501754y_{2} -0.719204y_{3} + 0.099562y_{4} \leq -0.052215, \\ \hphantom{\text{s.t.}\quad} 0.445225y_{1} -0.346896y_{2} + 0.637939y_{3} -0.257623y_{4} \leq 0.427920, \\ \hphantom{\text{s.t.}\quad} {-}0.202821y_{1} + 0.647361y_{2} + 0.920135y_{3} -0.983091y_{4} \leq 0.840950, \\ \hphantom{\text{s.t.}\quad} {-}0.886420y_{1} -0.802444y_{2} -0.305441y_{3} -0.180123y_{4} \leq -1.353686, \\ \hphantom{\text{s.t.}\quad} {-}0.515399y_{1} -0.424820y_{2} + 0.897498y_{3} + 0.187268y_{4} \leq 2.137251, \\ \hphantom{\text{s.t.}\quad} {-}0.591515y_{1} + 0.060581y_{2} -0.427365y_{3} + 0.579388y_{4} \leq -0.290987, \\ \hphantom{\text{s.t.}\quad} 0.423524y_{1} + 0.940496y_{2} -0.437944y_{3} -0.742941y_{4} \leq 0.373620, \\ \hphantom{\text{s.t.}\quad} y_{1} \geq 0, y_{2} \geq 0, y_{3} \geq 0, y_{4} \geq 0. \end{cases} $$

### Example 4

$$\begin{aligned} \textstyle\begin{cases} \min 6.5y_{1}-0.5y_{1}^{2}-y_{2}-2y_{3}-3y_{4}-2y_{5}-y_{6} \\ \text{s.t.}\quad y_{1}+2y_{2}+8y_{3}+y_{4}+3y_{5}+5y_{6}\leq 16, \\ \hphantom{\text{s.t.}\quad} {-}8y_{1}-4y_{2}-2y_{3}+2y_{4}+4y_{5}-y_{6}\leq -1, \\ \hphantom{\text{s.t.}\quad}2y_{1}+0.5y_{2}+0.2y_{3}-3y_{4}-y_{5}-4y_{6}\leq 24, \\ \hphantom{\text{s.t.}\quad}0.2y_{1}+2y_{2}+0.1y_{3}-4y_{4}+2y_{5}+2y_{6}\leq 12, \\ \hphantom{\text{s.t.}\quad} {-}0.1y_{1}-0.5y_{2}+2y_{3}+5y_{4}-5y_{5}+3y_{6}\leq 3, \\ \hphantom{\text{s.t.}\quad} 0\leq y_{i}\leq 10 , i=1,2,\ldots,6. \end{cases}\displaystyle \end{aligned}$$

### Example 5

$$\begin{aligned} \textstyle\begin{cases} \min 2y_{1}+3y_{2}-2y_{1}^{2}-2y_{2}^{2}+2y_{1}y_{2} \\ \text{s.t.}\quad {-}y_{1}+y_{2}\leq 1, \\ \hphantom{\text{s.t.}\quad}y_{1}-y_{2}\leq 1, \\ \hphantom{\text{s.t.}\quad} {-}y_{1}+2y_{2}\leq 3, \\ \hphantom{\text{s.t.}\quad}2y_{1}-y_{2}\leq 3, \\ \hphantom{\text{s.t.}\quad} 0\leq y_{1}\leq 15, 0\leq y_{2}\leq 15. \end{cases}\displaystyle \end{aligned}$$

### Example 6

$$\begin{aligned} \textstyle\begin{cases} \min {y^{T}Qy+c^{T}y} \\ \text{s.t.}\quad Ay\leq b, \\ \hphantom{\text{s.t.}\quad}y\in Y^{0}=\{0\leq y_{1} \leq 2,0\leq y_{2} \leq 2 \}, \end{cases}\displaystyle \end{aligned}$$

where $c=(2,4)^{T}, b=(1,2,4,3,1)^{T}, Q= \bigl ({\scriptsize\begin{matrix}{} -1 & 2 \cr 2 & -4 \end{matrix}} \bigr )$, $A= \left({\scriptsize\begin{matrix}{} -4 & 2 \cr 0 & 1 \cr 1 & 1 \cr 1 & 0 \cr 1 & -4 \end{matrix}} \right)$.

### Example 7

(Ref. [12])

$$\begin{aligned} \textstyle\begin{cases} \min -\sum_{i=1}^{n}y^{2}_{i} \\ \text{s.t.}\quad \sum_{i=1}^{j}y_{i}\leq j, j=1,2,\ldots,n, \\ \hphantom{\text{s.t.}\quad}y_{i}\geq 0, i=1,2,\ldots,n. \end{cases}\displaystyle \end{aligned}$$

Table 2 lists the numerical results of our algorithm, Gao’s algorithm [12], and Jiao’s algorithm [30] on Example 7. By comparing the numerical results in Table 2, we can conclude that our algorithm applied to Example 7 is superior to Gao’s algorithm [12] and Jiao’s algorithm [30] in terms of number of iterations and time.

Some randomly generated test examples with a large scale number of variables and constraints are used to validate the robustness of the proposed algorithm. These randomly generated examples and their computational results are given as follows.

### Example 8

$$\begin{aligned} \textstyle\begin{cases} \min y^{T}Qy+c^{T}y \\ \text{s.t.}\quad Ay\leq b, \\ \hphantom{\text{s.t.}\quad} y\in Y^{0}= \{ -10 \le y_{i} \le 10,i=1,2,\ldots,n \} , \end{cases}\displaystyle \end{aligned}$$

where $Q_{n\times n}$ is a real symmetric matrix, all the real elements of $Q_{n\times n}$, $A_{m\times n}$, and $c_{n\times 1}$ are randomly generated in Interval $[-2,2]$, all the real elements of $b_{m\times 1}$ are randomly generated in Interval $[1,10]$. For Example 8, we solved 10 different random instances for each size and presented statistics of the results. The computational results are summarized in Table 3, where the following notations have been used in column headers: Ave.Iter.: the average number of iterations; Ave.Time (s): the average CPU execution time for the algorithm in seconds; *m*: the number of constraints; *n*: the number of variables.

**Table 3:** Numerical results for Example 8

*n*	*m*	Ave.Iter.	Ave.Time (s)
5	5	1.2	0.5089
10	10	1.2	1.1423
15	10	1.3	4.7854
30	30	1.3	8.9569
40	40	1.2	8.4528
50	50	2.5	21.3985
80	80	2.4	33.3828
100	50	3.6	29.3468
100	100	5.4	64.5159
200	20	3.2	26.9366
200	50	3.2	42.3695
300	50	3.5	89.3652
300	100	4.8	436.2315

**Figure 1:**
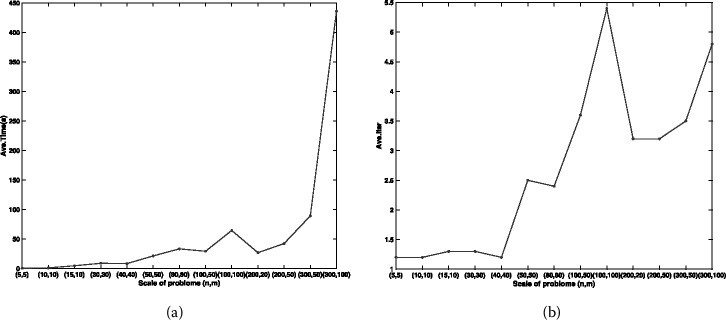
The variation tendency of performance index with scale of Example 8

From Table 3 and Fig. 1, it can be seen that, when *m* and *n* are below 50, the algorithm can find the global optimal solution in a short time and with lower iteration number. As the problem size becomes larger, the average number of iterations and the average CPU time of our algorithm are also increased, but they are not very sensitive to the problem size.

From the experimental results in Table 3, we can see that the proposed algorithm with the given convergent error can be used to globally solve the (NQP) with a large scale number of constraints and variables. The results in Tables 1–3 show that our algorithm is both feasible and efficient.

## Concluding remarks

In this paper, we propose a new branch-and-bound algorithm for globally solving the nonconvex quadratic programming problem. In this algorithm, we present a new linear relaxation method, which can be used to derive the linear programs relaxation problem of the investigated problem (NPQ). To accelerate the computational speed of the proposed branch-and-bound algorithm, an interval deleting rule is used to reduce the investigated regions. By subsequently partitioning the initial region and solving a sequence of linear programs relaxation problems, the proposed algorithm is convergent to the global optima of the initial problem (NPQ). Finally, compared with some existent algorithms, numerical results show higher computational efficiency of the proposed algorithm.
